# An Online Survey on the Current Trends in Root Canal Treatment

**DOI:** 10.7759/cureus.70348

**Published:** 2024-09-27

**Authors:** Ahmed Altuwalah

**Affiliations:** 1 Department of Restorative and Prosthodontic Dental Sciences, College of Dentistry, Majmaah University, Al Majmaah, SAU

**Keywords:** bioceramic, endodontic, general dentist, root canal treatment, survey

## Abstract

Root canal treatment in several regions of Saudi Arabia is commonly administered by general dental practitioners (GDPs) rather than specialized endodontists. The study aimed to compare the endodontic practices and preferences of general dental practitioners with those of endodontists and restorative specialists. A structured questionnaire including 23 questions focussing on fundamental principles and techniques used in contemporary root canal treatment was distributed online to 600 dentists who perform root canal treatments and are registered with the Saudi Dental Society (SDS). Data were gathered for basic demographic and professional details, practices, and preferences in endodontic materials and methods. The collected data were analyzed using SPSS version 21 (Armonk, NY: IBM Corp.). For selected variables, frequency distributions and 95% confidence interval for sample proportions were measured. Associations between categorical variables were examined using cross-tabulation, with statistical significance assessed by the chi-square test of homogeneity or Fisher’s exact test when necessary. The survey, with a 59% response rate primarily from participants aged 24-34 years (94.1%) with one to five years of experience (90.4%), revealed that most GDPs referred root canal treatment (RCT) cases due to lack of skills (82.8%), performed restorability assessments (90.9%), preferred Cavit for tooth buildup (87%), consistently used rubber dam isolation (91.5%), treated mainly anterior teeth (91.2%) without magnification (87.9%), used electronic apex locators for working length determination (63.3%), favored stainless steel K files and single cone obturation, while specialists exhibited wider clinical experience, more frequent use of advanced techniques, and higher usage of intracanal medicaments (91.7%) and ethylenediaminetetraacetic acid (EDTA) as irrigant (70.8%). The study provides valuable insights into the endodontic preferences and practices of dentists in the Kingdom of Saudi Arabia, revealing significant differences between general dental practitioners and specialists. The findings highlight critical areas for improvement, such as the need for enhanced training in endodontic skills and better access to modern materials and equipment.

## Introduction

Endodontic treatment, often referred to as root canal treatment, is an integral part of comprehensive and high-quality dental care. This procedure is designed to eliminate infection from the root canal system, prevent reinfection of the tooth, and save the natural tooth. Endodontic treatment is often the last resort to save a tooth before extraction becomes necessary. The importance of this procedure is underscored by the significant success rates reported in various studies [1-3]. Studies have shown that the success rates of root canal treatments performed by general dentists and specialists range between 74% and 90.83% [4-6].

The disparity in success rates highlights the critical role that specialized training and experience play in endodontic outcomes. Multiple endodontic organizations have established rigorous quality guidelines and standards for endodontic treatment [7,8]. These guidelines are continuously updated to reflect the latest advancements in our understanding of disease processes and technological innovations. For clinicians to provide the highest quality care, it is essential to stay abreast of current practices and continually refine their skills through lifelong learning [8].

Despite the existence of clear quality standards for healthcare providers, adherence to these guidelines remains inconsistent. Research conducted in various countries has revealed that the technical quality of root fillings and the adherence to recommended root canal treatment (RCT) protocols are often subpar [9-14]. This inconsistency can lead to variable treatment outcomes, potentially compromising patient care.

One of the critical components of successful endodontic treatment is the use of rubber dam isolation. This practice is considered the gold standard of care because it prevents contamination of the root canal system with saliva and other oral contaminants. A survey of American general dental practitioners revealed that only 59% reported consistent usage of rubber dams for isolation purposes [15]. This statistic is concerning, given the importance of rubber dam isolation in preventing infection and ensuring the success of root canal treatments.

The situation in Saudi Arabia presents additional challenges. Root canal treatment in several regions of Saudi Arabia is commonly administered by general dental practitioners (GDPs) rather than specialized endodontists. This is largely due to the limited availability and high cost of qualified endodontists. In contrast, GDPs are more readily accessible to patients and often serve as the primary providers of dental care [16]. However, numerous studies have indicated that GDPs do not consistently adhere to the recommended treatment guidelines. This deviation from best practices can result in lower-quality care and success rates for endodontic treatment that range from 65% to 75% [16-18]

This study aimed to gather comprehensive information on the technology and tools currently utilized by general practitioners in the Kingdom of Saudi Arabia for performing endodontic treatment. By comparing these practices with those of endodontists and restorative specialists, we aimed to identify gaps in knowledge, adherence to guidelines, and opportunities for improving the quality of care. By doing so, we hope to contribute to the ongoing effort to improve the quality of endodontic care and ensure that all patients receive the best possible treatment outcomes.

## Materials and methods

A structured questionnaire was developed and distributed to 600 dentists who perform root canal treatments and are registered with the Saudi Dental Society (SDS). The participants were selected randomly with the assistance of the SDS and its member lists. The inclusion criteria required that participants be dentists or specialists performing root canal treatments, practicing in Saudi Arabia, members of the SDS, and have an electronic email contact. Dentists who did not complete the survey were excluded from the study.

The questionnaire was anonymous and included 23 questions focused on the fundamental principles and techniques used in contemporary root canal treatment (appendix). The questions were designed to be closed-ended, emphasizing the use of new tools such as rotary instruments, magnification devices, and materials used for endodontic treatment. In the initial section of the questionnaire, participants provided anonymized demographic information, including gender, age, and years of working experience. This section also inquired about the early stages of RCT, such as access cavity preparation, pulp extirpation, tooth build-up, and temporization. The second section targeted staff who completed the entire RCT procedure, concentrating on the types of teeth treated, standard treatment protocols, and the utilization of technology and materials. The questionnaires were provided in both English and Arabic.

A pilot test was conducted with 10 dentists and endodontists to assess their understanding and the clarity of the questionnaire. Feedback from this pilot test was used to refine the questionnaire into its final version. The questions were designed to ensure unbiased responses, with participants selecting the most suitable answers from a provided list or writing their own if necessary. For certain questions, multiple selections were allowed.

The research was reviewed and approved by the Research Ethics Committee of Majmaah University. Online questionnaires were distributed via the "SurveyMonkey" web interface. Participants signed an informed consent form before enrolling in the study. All responses to the questionnaire were kept anonymous. Ultimately, 354 dental practitioners completed the questionnaires, resulting in a response rate of 59% (N=354).

Statistical analyses were conducted using SPSS version 21 (Armonk, NY: IBM Corp.). For selected variables, frequency distributions and 95% confidence interval for sample proportions were measured to indicate the expected range for a proportion in the reference population. Associations between categorical variables were examined using cross-tabulation, with statistical significance assessed by the chi-square test of homogeneity or Fisher’s exact test when necessary. A p-value of <0.05 was considered significant.

## Results

Basic demographic and professional details

The general characteristics of the participants are given in Table 1. Out of the 354 participants, 330 were general dental practitioners and 24 were endodontists or restorative specialists, herein referred to as specialists. Fifty-four percent (N=191) of the participants were male and 46% (N=163) were females. The majority of the participants belonged to the younger age group of 24-34 years (94.1%, N=333) with years of experience of one to five years (90.4%, N=320). The years of experience of the specialists varied widely compared to those of the general dental practitioners (Table 1).

**Table 1:** Characteristics of the participants. The general demographic distribution of the study population in terms of age, gender, and years of experience. The values were expressed as N, %, and 95% confidence interval. A p-value less than 0.05 is considered statistically significant.

Characteristics	Subgroup	Total participants (N=354)		General dental practitioner (N=330)		Specialist (endodontist/restorative specialist) (N=24)		p-Value
		N (%)	95% CI	N (%)	95% CI	N (%)	95% CI	
Age (years)	24-34	333 (94.1)	91.5-96.3	318 (96.4)	94.2-98.2	15 (62.5)	41.7-79.2	<0.001
	35-44	14 (4)	2.0-5.9	9 (2.7)	0.9-4.5	5 (20.8)	4.2-37.5	
	45-54	6 (1.7)	0.6-3.1	3 (0.9)	0.0-2.1	3 (12.5)	0-29.2	
	>65	1 (0.3)	0.0-0.8	0 (0)	-	1 (4.2)	0-12.5	
Gender	Male	191 (54)	48.3-59.3	181 (54.8)	49.7-60.3	10 (41.7)	20.8-58.3	0.289
	Female	163 (46)	40.7-51.7	149 (45.2)	39.7-50.3	14 (58.3)	41.7-79.2	
Years of experience	1-5	320 (90.4)	87.3-93.5	311 (94.2)	91.5-96.7	9 (37.5)	16.7-58.3	<0.001
	6-10	18 (5.1)	2.8-7.6	11 (3.3)	1.5-5.5	7 (29.2)	12.5-50	
	11-15	9 (2.5)	1.1-4.5	7 (2.1)	0.6-3.9	2 (8.3)	0-20.8	
	16-20	4 (1.1)	0.3-2.3	1 (0.3)	0.0-0.9	3 (12.5)	0-29.2	
	>20	3 (0.8)	0.0-2	0 (0)	-	3 (12.5)	0-25	

Initiation of root canal treatment by general dental practitioners

The responses by general dental practitioners to questions regarding the initiation of root canal treatment are given in Table 2. The majority of GDPs responded that they performed tooth restorability assessment (90.9%, N=300) and access cavity preparation (97.6%, N=322) by themselves. However, only 4.8% (N=16) of GDPs reported performing complete RCT for all cases, while 90.6% (N=299) referred some cases to specialists and 4.5% (N=15) referred all the RCT cases to specialists. The most commonly used material for tooth build-up before RCT referral was Cavit (87%, N=287) (Table 2).

**Table 2:** Initiation of root canal treatment by general dental practitioners. The responses related to the initiation of root canal treatment by general dental practitioners. The values were expressed as N, %, and 95% confidence interval.

Question	Response	N (%)	95% CI
Perform tooth restorability assessment for endodontic cases	Yes	300 (90.9)	87.6-94.2
	No (refer to a specialist to decide)	30 (9.1)	5.8-12.4
Perform access cavity preparation for endodontic treatment	Yes	322 (97.6)	95.8-99.1
	No	8 (2.4)	0.9-4.2
Material routinely used for tooth buildup before RCT referral	Cavit	287 (87)	83.3-90.6
	Composite restoration	3 (0.9)	0-2.1
	Glass ionomer cement	23 (7)	4.2-9.7
	Zinc oxide/eugenol cement	17 (5.2)	3.0-7.6
Perform RCT	No (refer all RCT cases to a specialist)	15 (4.5)	2.4-7.3
	Yes (all cases)	16 (4.8)	2.4-7.6
	Yes (refer some cases)	299 (90.6)	87.3-93.6

**Figure 1:**
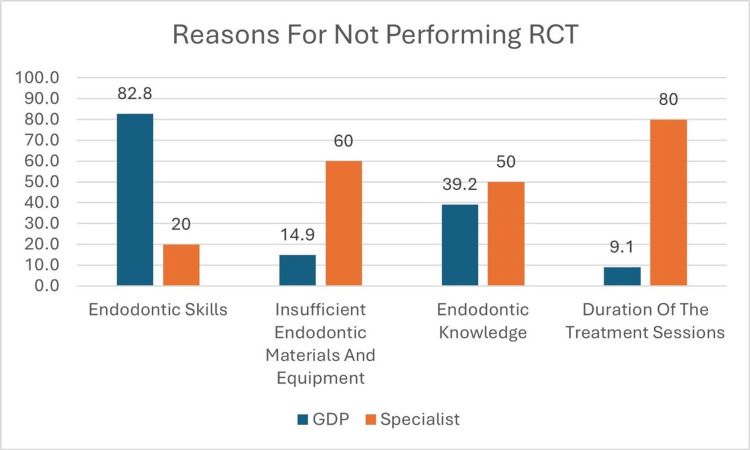
Reasons for not performing RCT. RCT: root canal treatment; GDP: general dental practitioners

**Figure 2:**
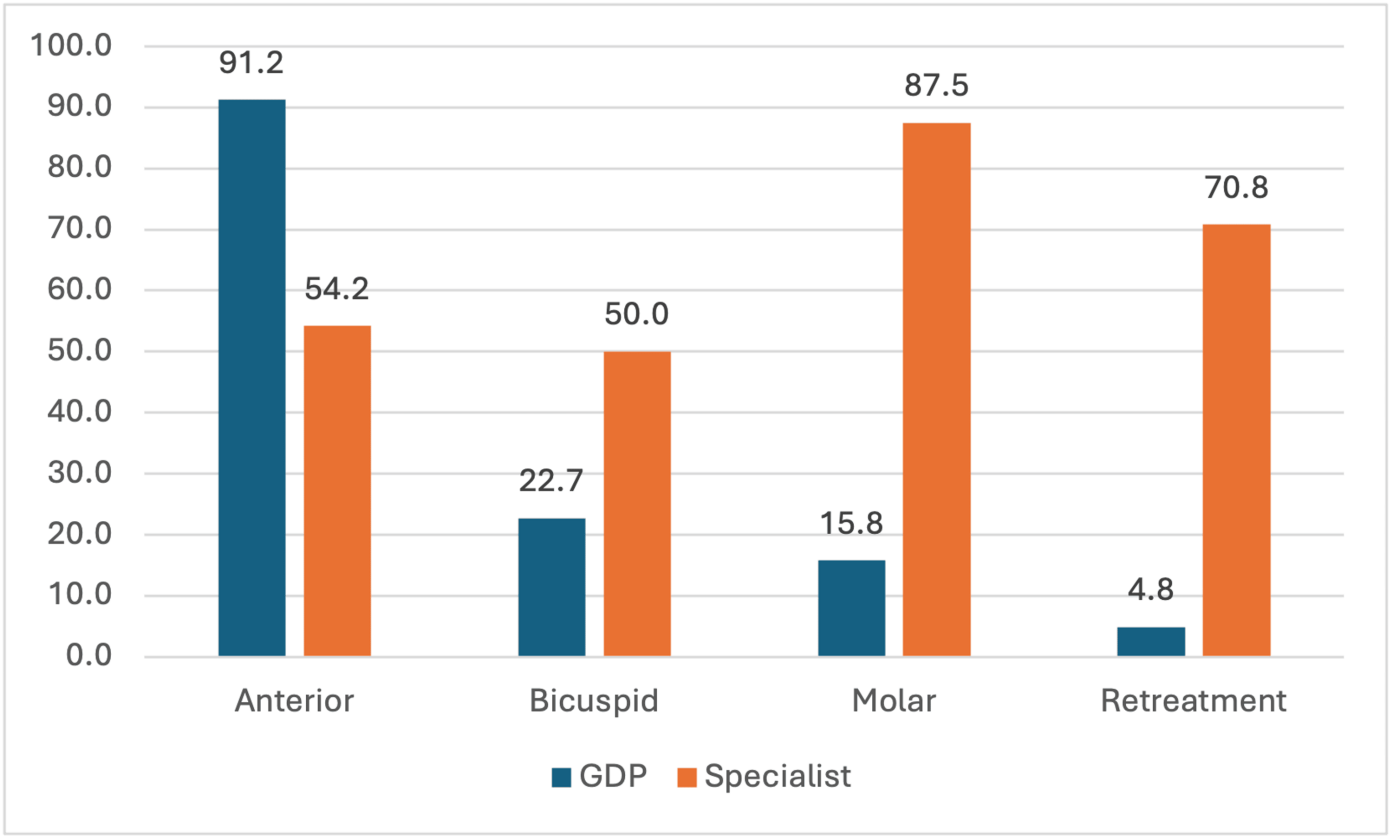
Types of teeth treated by general dental practitioners and specialists. GDP: general dental practitioners

Regarding reasons for not performing RCT, 82.8% (N=271) of GDPs reported lack of endodontic skills as the main reason and 80% (N=19) of specialists reported the longer duration of the treatment sessions to be the reason (Figure 1). GDPs treated 91.2% (N=300) of anterior teeth, 22.7% (N=73) of bicuspids, 15.8% (N=52) of molars, and 4.8% (N=16) of retreatments they encountered for RCT, and specialists reported to treat 54.2% (N=13) of anterior teeth, 50% (N=12) of bicuspids, 87.5% (N=21) of molars, and 70.8% (N=17) of retreatment cases they encountered (Figure 2).

Preference for root canal treatment practice by the participants

Isolation Methods

A total of 91.5% (N=324) of total participants used rubber dam isolation always in their practice. A negligible portion of GDPs (0.6%, N=2) reported to never have used rubber dam isolation. There was no statistically significant difference in the usage of rubber dams between GDPs and specialists (p=0.748) (Table 3).

**Table 3:** Root canal treatment: a comparison between general dental practitioners and specialists. The response from general dentists and specialists related to the practice of root canal treatment. The values were expressed as N, %, and 95% confidence interval. A p-value less than 0.05 is considered statistically significant.

Question	Response	Total participants (N=354)		General dental practitioner (N=330)		Specialist (endodontist/restorative specialist) (N=24)		p- Value
		N (%)	95% CI	N (%)	95% CI	N (%)	95% CI	
Frequency of using rubber dam isolation	Always	324 (91.5)	88.7-94.4	302 (91.5)	88.5-94.5	22 (91.7)	79.2-100	0.748
	Never	2 (0.6)	0.0-1.4	2 (0.6)	0.0-1.5	0		
	Sometimes	19 (5.4)	3.1-7.6	17 (5.2)	3.0-7.6	2 (8.3)	0.0-20.8	
	Usually	9 (2.5)	0.8-4.5	9 (2.7)	1.2-4.5	0		
Type of magnification used	I don’t use	298 (84.2)	80.2-87.9	290 (87.9)	84.2-91.2	8 (33.3)	16.7-54.2	<0.001
	Loupes	46 (13)	9.6-16.7	38 (11.5)	8.2-15.2	8 (33.3)	16.7-54.2	
	Microscope	10 (2.8)	1.1-4.5	2 (0.6)	.0-1.5	8 (33.3)	16.7-54.2	
Determining the working length	Electronic apex locator alone	215 (60.7)	55.4-65.8	209 (63.3)	57.9-68.8	6 (25)	8.3-41.7	<0.001
	Electronic apex locator with radiographic confirmation	77 (21.8)	17.2-26.3	61 (18.5)	14.2-22.7	16 (66.7)	45.9-83.3	
	Radiographs alone	62 (17.5)	13.8-21.7	60 (18.2)	14.2-22.7	2 (8.3)	0.0-20.8	
Hand instruments routinely used	H-files	70 (19.8)	15.5-24	68 (20.6)	16.1-24.8	2 (8.3)	0.0-20.8	<0.001
	NiTi Hand files	20 (5.6)	3.1-7.9	13 (3.9)	2.1-6.1	7 (29.2)	12.5-50	
	Stainless steel S K-files	264 (74.6)	69.8-78.5	249 (75.5)	70.9-80.3	15 (62.5)	41.7-79.2	
Obturation technique most commonly used	Carrier-based obturator	1 (0.3)	0.0-0.8	1 (0.3)	0.0-0.9	0	0	<0.001
	Cold lateral	28 (7.9)	5.4-10.7	22 (6.7)	3.9-9.4	6 (25)	8.3-41.7	
	Single cone	312 (88.1)	84.7-91.5	302 (91.5)	88.2-94.5	10 (41.7)	20.8-62.5	
	Warm vertical	13 (3.7)	2.0-5.6	5 (1.5)	0.3-3	8 (33.3)	16.7-54.2	
Type of sealer used	AH Plus sealer	291 (82.2)	78.0-86.2	279 (84.5)	80.9-88.2	12 (50)	29.2-70.8	<0.001
	Bioceramic sealer	42 (11.9)	8.8-15.5	33 (10)	7.0-13	9 (37.5)	20.8-58.3	
	Zinc oxide eugenol sealer	21 (5.9)	3.7-8.5	18 (5.5)	3.3-8.2	3 (12.5)	0.0-25	

Type of magnification

There was a statistically significant difference between GDPs and specialists in the usage of magnification for RCT (p<0.001). A total of 87.9% (N=290) of GDPs and 33.3% (N=8) of specialists reported to not use magnification. Surgical loupes were used by 11.5% (N=38) of GDPs and 33.3% (N=8) of specialists (Table 3).

Working length determination

The most preferred method of working length determination varied significantly between GDPs and specialists (p<0.001). GDPs mostly preferred the electronic apex locator alone (63.3%, N=209) while 66.7% (N=16) of specialists reported using electronic apex locator with radiologic confirmation (Table 3).

Instruments, sealer, and obturation technique

Stainless steel K files were the most popularly used hand instrument (74.6%, N=264) with 75.5% (N=249) of GDPs and 62.5% (N=15) of specialists preferring them. Statistically significant differences between GDPs and specialists were observed in the preference for routinely used hand instruments (p<0.001). There was a significant difference between GDPs and specialists in the type of sealer used in their RCT practice despite AH Plus sealer being the most popular one used (p<0.001). The single cone obturation technique was the commonly preferred technique by the participants (88.1%, N=312) and the Carrier-based obturator was the least favorite (0.3%, N=1). The preferred obturation technique showed a significant difference between GDPs and specialists (p<0.001) (Table 3).

Preference in root canal treatment factors by the participants

Seventy-five percent (N=18) of specialists used paste/gel type chelator/lubricant during canal instrumentation while only 20.6% (N=68) of GDPs did so which was statistically significant (p<0.001). Ethylenediaminetetraacetic acid (EDTA) as an irrigant was used by 70.8% (N=17) of specialists and 15.5% (N=51) of GDPs with significant difference (p<0.001). There was a significant difference between GDPs and specialists in the use of intracanal medicaments between RCT visits (p<0.001). The majority of the participants reported using sodium hypochlorite as the primary irrigant (96%, N=340) with no significant difference between GDPs and specialists (p=0.254). A total of 4.5% (N=15) of GDPs and 12.5% (N=3) of specialists left the tooth open for drainage during RCT. However, the difference was not statistically significant (p=0.087) (Table 4).

**Table 4:** A comparison between general dental practitioners and specialists in a range of treatment factors. The response from general dentists and specialists related to the practice about the range of treatment available for root canal treatment. The values were expressed as N, %, and 95% confidence interval. A p-value less than 0.05 is considered statistically significant. EDTA: ethylenediaminetetraacetic acid

Treatment factor	Response	Total participants (N=354)		General dental practitioner (N=330)		Specialist (endodontist/restorative specialist) (N=24)		p-Value
		N (%)	95% CI	N (%)	95% CI	N (%)	95% CI	
Routinely use a paste/gel type chelator/lubricant during canal instrumentation	Yes	86 (24.3)	20.1-28.5	68 (20.6)	16.4-25.2	18 (75)	58.3-91.7	<0.001
	No	268 (75.7)	71.5-79.9	262 (79.4)	74.8-83.6	6 (25)	8.3-41.7	
Using sodium hypochlorite as the primary irrigant	Yes	340 (96)	94.1-98	318 (96.4)	1.8-5.8	22 (91.7)	79.2-100	0.254
	No	14 (4)	2-5.9	12 (3.6)	94.2-98.2	2 (8.3)	0.0-20.8	
Using EDTA 17% as an irrigant	Yes	68 (19.2)	15-23.4	51 (15.5)	11.8-19.7	17 (70.8)	50-87.5	<0.001
	No	286 (80.8)	76.6-85	279 (84.5)	80.3-88.2	7 (29.2)	12.5-50	
Using intracanal medicaments between visits in cases with recurrent visits	Yes	92 (26)	21.2-30.2	70 (21.2)	17-25.5	22 (91.7)	79.2-100	<0.001
	No	262 (74)	69.8-78.8	260 (78.8)	74.5-83	2 (8.3)	0.0-20.8	
Leaving teeth open for drainage	Yes	18 (5.1)	2.8-7.6	15 (4.5)	2.4-7	3 (12.5)	0.0-25	0.087
	No	336 (94.9)	92.4-97.2	315 (95.5)	93-97.6	21 (87.5)	75-100	

## Discussion

The main aim of the study was to collect and compile information regarding endodontic preferences and practices of dentists in the Kingdom of Saudi Arabia. The response rate of the survey was 59% which is better than studies conducted in similar populations within the Kingdom of Saudi Arabia and globally [19-21]. Most of the survey participants belonged to the age group of 24-34 years (94.1%, N=333). Consequently, the majority had between 1 and 5 years of experience (90.4%, N=320), which contrasts with a similar study conducted in Qatar where the most common age group of participants was between 35 and 54 years [22]. However, in the current study, the specialists had a wider range of clinical experience when compared to GDPs.

In the current study, only a small percentage of general dental practitioners (4.8%, N=16) performed all of their RCT cases themselves. In contrast, 90.6% (N=299) reported referring some cases to specialists, while 4.5% (N=15) referred all of their RCT cases. The reasons reported for this were mainly a lack of endodontic skills (82.8%, N=273) and endodontic knowledge (39.2%, N=129). The less common reasons mentioned were insufficient endodontic materials and equipment (14.9%) and duration of the treatment sessions (9.1%, N=30). However, among the specialists, the most frequently reported reasons for referring their RCT cases were the duration of the treatment sessions (80%, N=19) and insufficient endodontic materials and equipment (60%, N=14). As shown in earlier studies, dentists' decisions about restorative treatments are influenced by various factors, including age, preventive mindset, knowledge, and obstacles [23-25]. Studies show that the decision-making process for restorations involves a complex interaction of clinical expertise, the type of patient practice, as well as biological and environmental considerations [26,27].

In this study, 90.9% (N=300) of GDPs performed tooth restorability assessment and 97.6% (N=322) of GDPs performed access cavity preparation for endodontic treatment. The assessment of tooth restorability by general dental practitioners and specialists is crucial for treatment planning. Access cavity preparation in endodontics is essential for successful root canal treatment, as it ensures proper visibility, access, and disinfection while preserving tooth structure and increasing resistance to fracture [28-30]. Modern techniques, such as conservative endodontic cavity preparation and image-guided therapies, prioritize minimizing tooth structure removal, thereby enhancing tooth strength and promoting better long-term outcomes [30,31]. Appropriate case selection is crucial for the success of endodontic treatment [32]. Studies have shown that dental interns and freshly graduated dentists face challenges in making restorability decisions, raising concerns about the adequacy of learning outcomes and graduate attributes [33].

Tooth buildup before referral for root canal treatment is essential to restore structural integrity and provide adequate support during the procedure. This preparation also enhances the effectiveness of the endodontic treatment by ensuring a stable environment for the dentist [34,35]. Cavit (87%, N=287) was the commonly preferred material for tooth buildup before RCT referral by the GDPs. Seven percent (N=23) used glass ionomer cement and 5.2% (N=17) used zinc oxide eugenol cement.

A clinically significant finding in the current study is the stringent adherence of the dentists of Saudi Arabia to the standard of care in the use of rubber dam isolation. A total of 91.5% (N=302) and 91.7% (N=22) of GDPs and specialists always used rubber dam isolation. Only 0.6% (N=2) of GDPs reported to never have used rubber dam isolation. This result is in contrast to earlier studies conducted in other countries like Qatar [22], Hong Kong [36], the United States [13], and the United Kingdom [14], where reduced compliance to using rubber dams for RCT cases was observed in GDPs.

Regarding the type of teeth treated by the dentists for RCT, GDPs mainly treated RC of the anterior teeth (91.2%, N=301) while specialists mostly treated RC of molars (87.5%, N=21) as well as retreatment cases (70.8%, N=17) (Figure 2). This result is similar to the study conducted in Qatar by Alemam et al. in 2024 [22]. Anterior teeth with single root is relatively easier to treat when compared to molars with two or more root canals and hence require better endodontic skill and knowledge which precisely were reasons given by the GDPs in our study for referring their RCT cases.

In the current study, there was a significant difference in the use of magnification for RCT between GDPs and specialists. A total of 87.9% (N=290) of GDPs and 33.3% (N=8) of specialists reported never using magnification for RCT. Surgical loupes were used by 11.5% (N=38) of GDPs and 33.3% (N=8) of specialists. Similar differences were observed by Alemam et al. in 2024 [22]. A recent study conducted in the Kingdom of Saudi Arabia found that magnification devices were commonly used by GDPs and specialists in the Kingdom and 72% reported that the use of magnification devices during RCT improved quality of life [37]. Though there is a lack of strong evidence that suggests that the use of magnification could improve root canal treatment outcomes, there is no denying that the use of magnification in locating root canals significantly improves the accuracy of root canal treatment and enhances the dentist's ability to identify complex canal systems, reducing the likelihood of missed canals and treatment failure [38,39].

The current study found a significant difference between GDPs and specialists in the working length determination procedure with GDPs (63.3%, N=209) mainly preferring the electronic apex locator alone, while 66.7% (N=220) of specialists preferred electronic apex locator with radiographs for working length determination. This contrasts with the study conducted in Qatar which did not find any significant difference [22]. There is no strong evidence to support the efficacy of electronic apex locators compared to radiographs and individual preferences could play a role in the current result [40,41].

Stainless steel K-files were the commonly preferred hand instrument by both GDPs and specialists, which is similar to the study in Qatar [22]. Single cone obturation technique was routinely preferred by GDPs and specialists in this study while cold lateral technique was preferred by dentists in Qatar [22]. The current study found a significant difference in the preference of hand instrument used and obturation technique between GDPs and specialists while the difference was not significant in the study done in Qatar [22]. EDTA as an irrigant was used by 70.8% (N=17) of specialists and only 15.5% (N=51) of GDPs, while a higher percentage of specialists (90%) and GDPs (49%) in Qatar used EDTA during RCT.

Using intracanal medicaments between visits in cases requiring multiple appointments helps to manage infection and promote healing in the root canal system. This practice ensures a conducive environment for successful endodontic treatment and reduces the risk of reinfection. In the current study, only 21.2% (N=70) of GDPs used intracanal medicaments between visits as opposed to 91.7% (N=22) of specialists. The use of intracanal medicaments is a dynamic process influenced by the practitioner's expertise, the complexity of the case, and the patient's response to treatment [1].

This study highlighted several challenges in endodontic practice among GDPs and specialists in Saudi Arabia and provided the following suggestions to address these issues. (1) Many GDPs reported insufficient skills (82.8%, N=273) and knowledge (39.2%, N=129), suggesting that additional training and continuing education programs are needed to enhance their capabilities. (2) Inadequate resources were another challenge, with GDPs (14.9%, N=49) and specialists (60%, N=14) both experiencing difficulties. Improving access to modern endodontic materials and equipment through better funding and resource allocation could address this issue. (3) Long treatment times were a significant concern for both GDPs (9.1%, N=30) and specialists (80%, N=19). Streamlining treatment protocols and enhancing efficiency through better time management and advanced techniques might help alleviate this problem. (4) The low usage of intracanal medicaments by GDPs (21.2%, N=70) compared to specialists (91.7%, N=22) could be improved by educating GDPs on the benefits and application of these medicaments through targeted training sessions. (5) Although there is high compliance with rubber dam isolation in Saudi Arabia, maintaining high standards is crucial, which can be achieved through regular audits and emphasizing the importance of rubber dam isolation in global training programs.

Strengths and limitations

The study achieved a high response rate of 59% (N=354), higher than similar studies both locally and globally. It gathered comprehensive data from a significant sample of dentists, provided detailed demographic information, and included comparisons with studies from other regions, offering a broader context. The study effectively identified key challenges faced by general dental practitioners (GDPs) and specialists, highlighted the high adherence to rubber dam isolation, and covered a wide range of clinical practices. However, there are some limitations to the study that need to be addressed. Most participants were young (24-34 years) with limited experience (one to five years), potentially not representing the broader dental practitioner population. The reliance on self-reported data may introduce bias and inaccuracies. Some findings, such as the use of magnification devices and electronic apex locators, are based on individual preferences rather than strong evidence, limiting generalizability. The study's focus on Saudi Arabia, despite comparisons with other regions, may not fully apply to countries with different healthcare systems and educational backgrounds. There is also a potential for response bias, as participants who chose to respond might have different views and practices compared to non-respondents.

## Conclusions

The study provides valuable insights into the endodontic preferences and practices of dentists in the Kingdom of Saudi Arabia, revealing significant differences between general dental practitioners (GDPs) and specialists. Despite a relatively young and less experienced participant demographic, the findings highlight critical areas for improvement, such as the need for enhanced training in endodontic skills and better access to modern materials and equipment. The high adherence to rubber dam isolation and the comprehensive coverage of various clinical practices underscores the commitment to maintaining high standards of care. Addressing the identified challenges through targeted education and resource allocation can further elevate the quality of endodontic practice in Saudi Arabia, ultimately improving patient outcomes.

## Appendices

**Table 5:** An online survey on the current trends in root canal treatment questions. EDTA: ethylenediaminetetraacetic acid; RCT: root canal treatment

Section 1: General																
Questions	Options															
1.1. Age (years)	18-24		25-34			35-44			45-54				55-64			>65
1.2. Gender	Male						Female									
1.3. Clinical role	Dentist		Restorative specialist								Endodontist					
1.4. How many years have you been working?	1-5		6-10			11-15			16-20				>20			
1.5. How many hours of endodontic CPD have you taken in the last two years?	None		1-5			6-10			11-15				16-20			>20
1.6. Do you do tooth restorability assessments for endodontic cases?	Yes					No (refer to specialist to decide)										
1.7. Do you perform access cavity preparation?	Yes							No								
1.8. What instrument do you mainly use during pulp extirpation procedure?	K-file	H-file			Barbed broach only							Pulpotomy (removal of pulp tissue from pulp chamber)				
1.9. What material do you routinely use before RCT case referral?	Zinc oxide/eugenol cement	Glass ionomer cement						Composite restoration								
1.10. Do you perform RCT?	Yes (all cases)			Yes (refer to some cases)						No (refer all RCT cases to specialist)						
1.11. If you do not perform RCT, what is your main reason(s)? Check all that apply.	Endodontic Knowledge			Endodontic skills						Duration of the treatment sessions						
Section 2 (only for clinicians who perform RCT)																
Questions	Options															
2.1. Which types of teeth do you routinely treat?	Anterior			Bicuspid						Molar				Retreatment		
2.2. How often do you use rubber dam isolation?	Always			Usually						Never				If you do not use a rubber dam, please state why.		
2.3. Do you use magnification?	No			Loupes						Microscope				Others		
2.4. How do you determine the working length?	Radiographs alone			Electronic apex locator alone						Electronic apex locator with radiographic confirmation						
2.5. What hand instrument do you routinely use during instrumentation?	Stainless steel S K-files			H-files						NiTi Hand files						
2.6. Do you routinely use a paste/gel type chelator/lubricant during canal instrumentation?	Yes									No						
2.7. Do you use sodium hypochlorite as your primary irrigant?	Yes									No						
2.8. Do you use EDTA 17% as an irrigant?	Yes									No						
2.9. In multiple visits cases, do you use intracanal medicaments between visits?	Yes									No						
2.10. Do you leave teeth open for drainage?	Yes									No						
2.11. What obturation technique do you most commonly use?	Single cone			Cold lateral						Warm vertical compaction					Carrier-based obturator	
